# A Suggested Diagnostic Approach for Sporadic Anthrax in Cattle to Protect Public Health

**DOI:** 10.3390/microorganisms9081567

**Published:** 2021-07-23

**Authors:** Jana Avberšek, Jasna Mićunović, Vasilij Cociancich, Tomislav Paller, Darja Kušar, Urška Zajc, Matjaž Ocepek, Silvio Špičić, Sanja Duvnjak, Mateja Pate

**Affiliations:** 1 Institute of Microbiology and Parasitology, Veterinary Faculty, University of Ljubljana, Gerbičeva ulica 60, 1000 Ljubljana, Slovenia; jasna.micunovic@vf.uni-lj.si (J.M.); darja.kusar@vf.uni-lj.si (D.K.); urska.zajc@vf.uni-lj.si (U.Z.); matjaz.ocepek@vf.uni-lj.si (M.O.); mateja.pate@gmail.com (M.P.); 2 National Veterinary Institute, Veterinary Faculty, University of Ljubljana, Gerbičeva ulica 60, 1000 Ljubljana, Slovenia; vasilij.cociancich@vf.uni-lj.si (V.C.); tomislav.paller@vf.uni-lj.si (T.P.); 3 Croatian Veterinary Institute, Savska cesta 143, 10000 Zagreb, Croatia; spicic@veinst.hr (S.Š.); marjanovic@veinst.hr (S.D.)

**Keywords:** anthrax, cattle, diagnostics, multiple-locus variable-number tandem repeat analysis (MLVA), public health, real-time PCR

## Abstract

The repeated occurrence of anthrax in grazing animals should be a reminder of a widespread presence of *Bacillus anthracis* spores in the environment. Its rapid diagnosis is critical to protect public health. Here, we report a case of anthrax in cattle that was investigated using conventional and molecular methods. In 2015, six cows suddenly died within three days and the number of dead animals increased to a total of 12 within two weeks. At necropsy, anthrax was suspected. Therefore, spleen tissue samples were collected (from 6/12 animals) and laboratory tests (microscopy, cultivation, and real-time PCR) performed. The results of tissue staining for microscopy and cultivation were in congruence, while *B. anthracis* real-time PCR outperformed both. Spleen tissues from all six animals were real-time PCR-positive, while *B. anthracis* was successfully cultivated and detected by microscopy from the spleen of only three animals. Additionally, the ear tissue from another (1/12) cow tested positive by real-time PCR, supporting the suitability of ear clippings for molecular confirmation of *B. anthracis*. Genotyping of the isolates using multiple-locus variable-number tandem repeat analysis (MLVA) revealed a common source of infection as all three typed isolates had an indistinguishable MLVA genotype, which has not been observed previously in Europe. The results indicate that molecular testing should be selected as the first-line tool for confirming anthrax outbreaks in animals to ensure timely protection of public health.

## 1. Introduction

Anthrax, an ancient zoonosis caused by *Bacillus anthracis*, is distributed globally and is enzootic in many regions of the world, especially in Asia, sub-Saharan Africa, and Central and South America [1]. The true worldwide incidence of anthrax is not known; however, epizootics occur each year, resulting in the death of hundreds to thousands of animals and transmission of the disease to humans. It is estimated that between 2000 and 20,000 human anthrax cases occur worldwide yearly [2,3].

Anthrax is not a major human or animal health issue in developed countries; in Europe, it is a rare disease with only a few cases reported annually. Between 2007 and 2019, 97 confirmed human anthrax cases were reported in Europe, ranging from one to 32 cases per year [4]. Animal anthrax cases were reported in Italy, Croatia, and Romania in 2020 and 2021 [5]. In Slovenia, the last human anthrax case dates back to 1983 (Maja Sočan, personal communication). Since then, fewer than ten animal cases have been confirmed in Slovenia, with the last case described here in 2015.

Rapid diagnosis of anthrax is necessary to prevent the spread of the bacteria. Several conventional and molecular microbiological methods for the detection of *B. anthracis* are available [6], but diagnostic algorithms vary worldwide from necropsy to various laboratory methods. Selecting the most appropriate methods is challenging, especially in countries where sporadic anthrax cases occur only every few decades. Here, we describe the most recent animal anthrax cases in Slovenia and compare the performance of methods available in our laboratory.

## 2. Materials and Methods

In August 2015, 6 cows from farm A were found dead in a marshland pasture in the central part of Slovenia. As a part of the national disease surveillance activities, 2 carcasses were sent to the National Veterinary Institute (NVI) laboratory for necropsy. In the following 2 weeks, another 6 cows from 4 farms (A–D) died in a nearby pasture; 4 carcasses, each from a different farm, were transported separately to the NVI laboratory. Therefore, samples from 6/12 dead cows (1/A, 2/A, 4/B, 5/A, 6/C and 7/D in Table 1) were examined for the presence of *B. anthracis* using laboratory tests, namely, microscopy of the stained spleen tissue, cultivation, and real-time PCR. Additionally, a clipped piece of an ear from another (1/12) cow was collected and subjected to real-time PCR (3/A in Table 1).

Spleen tissue smears were stained with methylene blue (Becton Dickinson, Franklin Lakes, NJ, USA) and examined under a light microscope for the presence of encapsulated *B. anthracis* cells. Samples were inoculated onto 5% sheep blood agar plates (Columbia Blood Agar Base, Oxoid by Thermo Fischer Scientific, Hampshire, UK) and incubated at 37 °C for 24 h. Suspect and ambiguous colonies (based on morphology) were tested using *B. anthracis* specific real-time PCR assay as described below for the tissue samples. DNA from bacterial colonies was extracted using a rapid lysis method (boiling of cell suspensions for 15 min, followed by centrifugation for 2 min at 14,000× *g* and filtration [0.45 μm membrane filter] of the supernatant). DNA from tissue samples (spleen, ear clipping) was extracted using a commercial kit (DNA Isolation from Complex Samples, Institute of Metagenomics and Microbial Technologies, Ljubljana, Slovenia), following the manufacturer’s instructions. The protocol included bead-beating (45 s at 6400 rpm) for 3 times using a tissue homogenizer (MagNA Lyser Instrument, Roche, Basel, Switzerland), combined with enzymatic and heat-induced lysis between mechanical shearing. A previously described and validated real-time PCR assay targeting the *capC* and *pag* genes of fully pathogenic *B. anthracis* was employed for DNA amplification (both for tissue samples and suspect colonies) [7], which was interpreted as an indication of the presence of *B. anthracis* because the result was supported by pathological and epidemiological data. Briefly, the reaction mix for each gene contained 10 µL of 2× master mix (TaqMan Fast Universal PCR Master Mix, Applied Biosystems by Thermo Fisher Scientific, Foster City, CA, USA), 1 µL of primer-probe mix with final concentrations of 900 nM for each primer and 250 nM for the probe, 2 µL of template DNA, and PCR-grade water to the final volume of 20 µL. PCR amplification (20 s at 95 °C, followed by 40 cycles of 3 s at 95 °C and 30 s at 60 °C) and amplicon detection were performed in a thermocycler 7500 Fast Real-Time PCR System (Applied Biosystems by Thermo Fisher Scientific).

Three obtained *B. anthracis* isolates (designated 1/A, 5/A, and 6/C in Table 1) were subjected to multiple-locus variable-number tandem repeat analysis (MLVA). Subsequently, an additional *B. anthracis* isolate (271/15) obtained 3 months later from a diseased cow, located approx. 50 km from the initial anthrax cases, was also MLVA-genotyped. MLVA was performed on 8 loci (*vrrA*, *vrrB*_1_, *vrrB*_2_, *vrrC*_1_, *vrrC*_2_, CG_3_, pXO1, pXO2) as described by Keim et al. [8]. For MLVA, DNA from bacterial colonies was isolated using the commercially available QIAcube DNA Mini Kit and the QIAcube system (Qiagen, Hilden, Germany) according to the manufacturer’s instructions. The reaction mix for each MLVA locus contained 10 µL of 2× master mix (HotStarTaq Master Mix, Qiagen), 0.5 μM of each primer pair specific for the target locus, 2 μL of template DNA, and PCR-grade water to the final volume of 20 µL. PCR amplification was adopted from Keim et al. [8] and performed in a thermocycler ProFlex PCR System (Applied Biosystems by Thermo Fisher Scientific). Amplicons were analyzed by QIAxcel capillary electrophoresis (Qiagen) using the QIAxcel DNA High Resolution Kit, QX Alignment Marker 15–5 kb, QX Size Marker 100 bp–2.5 kb, and OM1700 separation method. From MLVA results, amplicon lengths were converted into the number of individual repeats according to Keim et al. [8]. The number of individual repeats was further confirmed by Sanger sequencing of PCR amplicons (Macrogen, Amsterdam, The Netherlands). The results were presented as 8-digit numerical codes. The obtained codes were analyzed by applying the categorical coefficient to construct a minimum spanning tree using BioNumerics version 8.0 (bioMérieux, Applied Maths NV, Sint-Martens-Latem, Belgium). Data were compared with results from different countries deposited in the public *B. anthracis* v4_1 database (available at https://microbesgenotyping.i2bc.paris-saclay.fr/databases/view/9 (accessed on 18 June 2021)).

## 3. Results

Necropsy of the first two bloated cows examined (1/A and 2/A in Table 1) revealed rapid decomposition and bleeding from the nose and inner corners of the eyes. The blood was dark and unclotted, the spleen was severely congested and enlarged, and hemorrhagic content in the abomasum and small intestine was noted. The lungs were moderately edematous and the diaphragm was swollen. To avoid further contamination of the necropsy room and for biosafety reasons, samples from the other four suspect animals were harvested directly through an incision in the upper left corner of the abdominal wall.

In the spleen samples from 6/12 animals subjected to laboratory tests, the presence of *B. anthracis* was confirmed by at least one method: real-time PCR was positive in all six samples, in contrast to microscopy and cultivation, where only three samples (1/A, 5/A, and 6/C) were positive (Table 1). Staining results (microscopy) were in accordance with cultivation. Suspect bacterial colonies were obtained also from the spleen sample of animal 4/B, but *B. anthracis* was not confirmed by real-time PCR. Because cultivation yielded inconclusive results, additional samples from the small intestine and blood of animal 4/B were examined; cultivation of *B. anthracis* from the small intestine failed, but ambiguous results were again noticed for the blood sample (real-time PCR identification of colonies was negative for *B. anthracis*). In addition, real-time PCR was performed for the blood sample after DNA extraction, and it was positive for *B. anthracis*, but only *pag* was detected. The threshold cycle (Ct) value for the *pag* gene was 34.91, indicating a low *B. anthracis* load in the blood sample. In general, the Ct values for the *pag* gene were lower than for the *capC* gene (Table 1), which could be the reason for the observed negative *capC* result.

To inspect the suitability of a non-invasive sampling method for molecular confirmation of anthrax, a clipped piece of an ear from 1/12 dead cows (3/A) was collected and subjected to real-time PCR, which was positive for *B. anthracis* (Table 1, see Note 2). The Ct values obtained were the second lowest compared to the other six positive (spleen) samples, indicating a high *B. anthracis* load in the ear clipping.

**Figure 1:**
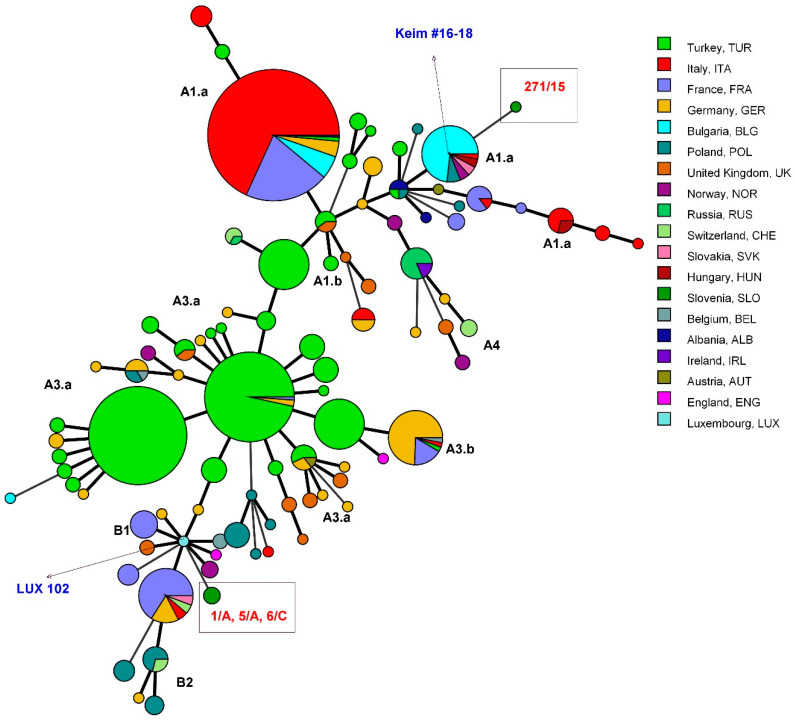
Minimum-spanning tree of *B. anthracis* multiple-locus variable-number tandem repeat analysis (MLVA) genotypes, constructed in BioNumerics using a categorical coefficient for clustering. The strains are marked with different colors according to the country of origin (see the legend); they originate from Slovenia (this study) or publicly available MLVA database (https://microbesgenotyping.i2bc.paris-saclay.fr/databases/view/9). The branches reflect the relationships between the strains (the thicker the line, the closer the genotypes). Slovenian strains are in the rectangles. The strain 102 from Luxembourg is marked as being the closest to the Slovenian strains 1/A, 5/A, and 6/C, and the genotypes 16–18 defined by Keim et al. [8] as one of the genotypes closest to the Slovenian strain 271/15. A and B labels refer to the genotype groups and subgroups defined by Keim et al. [8] to enable comparison; strains 1/A, 5/A, and 6/C belong to the genotype B.

All three isolates (1/A, 5/A, and 6/C) belonged to the same genotype, while the isolate 271/15 differed from the other typed isolates (Table 2). All Slovenian isolates had a unique MLVA genotype in comparison to the other European *B. anthracis* strains (Figure 1).

## 4. Discussion

Anthrax is a rare disease in Slovenia and Europe, but a prompt response to outbreaks in animals is crucial to minimize the risk of zoonotic transmission. One of the prerequisites for timely outbreak investigation is rapid and effective disease detection and confirmation, based on expert personnel of various specializations, from field veterinarians to pathologists and microbiologists. In the outbreak described here, the disease was confirmed in the laboratory within one day, and measures to prevent the spread of anthrax were immediately implemented on farms and pastures, including disinfection, animal movement bans, and vaccination. The State Centre for Disease Control defined a new anthrax district, an area where vaccination of all susceptible animals, grazing or receiving feed from the district, is mandatory for the next 50 years. All persons who came in contact with the dead and suspect animals were instructed to seek medical attention and were given antibiotic prophylaxis. 

The results of the outbreak investigation presented here indicate that molecular detection of *B. anthracis* in spleen tissue by real-time PCR should be considered as the method of choice for rapid confirmation of anthrax, as only 3/6 cows were found positive by staining and cultivation, but 6/6 by real-time PCR. In addition, the positive real-time PCR result for the ear sample indicates the suitability of ear clippings for rapid real-time PCR confirmation of *B. anthracis* without necropsy. In comparison to conventional PCR, real-time PCR is more sensitive [9]. Similarly to our case report, the superiority of PCR compared to cultivation and microscopy for the detection of *B. anthracis* in blood smears has also been reported [10]. In the latter study, PCR yielded positive results even from blood smears with degraded capsules that led to false-negative staining results. In conclusion, classical bacteriological methods appear as useful to complement the molecular methods to obtain *B. anthracis* isolates for subsequent genotyping purposes, such as MLVA or whole-genome sequencing (WGS). In our case, MLVA confirmed that the observed anthrax cases in cows in August 2015 were part of an outbreak, as all three typed isolates showed the same genotype, which was unique among other *B. anthracis* strains from Europe. The proposed diagnostic algorithm for anthrax outbreaks should therefore include real-time PCR as a first-line tool for the confirmation of *B. anthracis*, followed by genotyping of the obtained isolates to delineate the outbreak. 

In the present study, sheep blood agar plates were used for cultivation of *B. anthracis* from spleen tissue after necropsy, although PLET (polymyxin–lysozyme–EDTA–thallous acetate) agar is recommended when anthrax is suspected [11]; it is a suitable selective medium for contaminated samples but is not routinely used in our laboratory due to the small number of anthrax cases. Not using the PLET agar plates could represent a disadvantage in the efficient cultivation of *B. anthracis* in countries where anthrax occurs sporadically. However, in our case, contamination did not significantly hinder the isolation of *B. anthracis*, as *Proteus* sp. swarmed across the agar plate in only one sample. Although several chromogenic and selective agars are known for the detection of *B. anthracis* [12,13], maintaining their (including PLET agars) supply is difficult and expensive in laboratories that cover only a few anthrax cases every few years. Another issue for laboratories faced with the diagnosis of sporadic anthrax is the use of M’Fadyean alternative commercial methylene blue stains, which often give mixed results and lead to diagnostic failures [14]. 

In countries with regular anthrax cases, outbreaks may occur in very remote or challenging environments, and the samples collected may not be suitable for cultivation when they reach the laboratory [15]. Therefore, in countries with both endemic and sporadic anthrax, real-time PCR should be a preferred method for detecting *B. anthracis* in tissue samples. We collected spleen tissue at necropsy, but blood samples also proved suitable [10]. However, in our study, spleen samples were clearly superior to blood and intestinal samples collected *postmortem*. On the other hand, the non-invasively collected ear clipping sample performed well with real-time PCR, like the skin samples showing high sensitivity and specificity in a previous study [15]. Because anthrax is a fatal disease with a high mortality rate in humans and animals if not diagnosed and treated in time, rapid and reliable diagnosis is of utmost importance.

## Figures and Tables

**Table 1:** Investigation of anthrax outbreak in cattle: methods used and results obtained for the spleen samples.

Animal/Farm ^1^	Microscopy	Cultivation	Real-Time PCR (Ct for *capC*/*pag* Gene)
1/A	pos	pos	pos (16.37/15.51)
2/A	neg	neg	pos (21.72/19.72)
3/A ^2^	ND	ND	ND ^2^
4/B	ND	neg ^3^	pos (22.79/19.12)
5/A	pos	pos	pos (25.65/23.34)
6/C	pos	pos	pos (26.55/24.46)
7/D	neg	neg	pos (29.06/26.40)

^1^ Arranged chronologically as received for necropsy. ^2^ Only the ear-clipping sample was collected (not spleen) and analyzed by real-time PCR, which was positive for *B. anthracis* (Ct was 18.97 for *capC* and 16.73 for *pag*). ^3^ Suspect colonies were observed, but colony-confirmative real-time PCR was negative. Ct—threshold cycle value, ND—not determined (analysis not performed), neg—negative for *B. anthracis*, pos—positive for *B. anthracis.*

**Table 2:** Results of multiple-locus variable-number tandem repeat analysis (MLVA).

	MLVA Loci (Length (bp)/Number of Repeats)															
Isolate Name	*vrrA*		*vrrB*_1_		*vrrB*_2_		*vrrC*_1_		*vrrC*_2_		CG3		pXO1		pXO2	
1/A	301	3	184	15	162	14	613	57	532	17	158	2	117	5	133	6
5/A	301	3	184	15	162	14	613	57	532	17	158	2	117	5	133	6
6/C	301	3	184	15	162	14	613	57	532	17	158	2	117	5	133	6
271/15	325	5	229	20	162	14	649	63	586	20	153	1	132	10	137	8

## Data Availability

The data presented in this study are available on reasonable request from the corresponding author.

## Abbreviations

Ct, threshold cycle value (in real-time PCR); MLVA, multiple-locus variable-number tandem repeat analysis; NVI, National Veterinary Institute; PLET agar, polymyxin–lysozyme–EDTA–thallous acetate agar; WGS, whole-genome sequencing.
